# Initiating disease-modifying treatments in multiple sclerosis: Measuring the decision process using decisional conflict and decisional regret scales

**DOI:** 10.1177/2055217319833006

**Published:** 2019-02-27

**Authors:** DD Wilkie, A Solari, R Nicholas

**Affiliations:** Imperial College, Centre for Neuroscience, Imperial College Faculty of Medicine, London, UK; Imperial Healthcare NHS Trust, Charing Cross Hospital, Fulham Palace Rd, UK; Unit of Neuroepidemiology, Fondazione IRCCS Istituto Neurologico C. Besta, Milan, Italy; Imperial College, Centre for Neuroscience, Imperial College Faculty of Medicine, London, UK; Imperial Healthcare NHS Trust, Charing Cross Hospital, Fulham Palace Rd, UK; Centre for Neuroinflammation and Neurodegeneration, Faculty of Medicine, Imperial College London, London, United Kingdom

**Keywords:** Multiple sclerosis, decision-making, shared decision making, disease-modifying therapies

## Abstract

**Introduction:**

Initiating disease-modifying treatments (DMTs) in multiple sclerosis (MS) is a major decision for people with (pw)MS but little is known about how the decision is perceived by the individual.

**Objectives:**

The aim of the study was to determine if decisional conflict (DC) and decisional regret reflect different stages of the decision-making process when initiating DMTs.

**Methods:**

This was a cross-sectional study of three cohorts of pwMS (*n* = 254), a ‘MS conference attendees’, ‘on treatment’ and an ‘offered treatment’ cohort. Questionnaires assessing DC, decisional regret and control preference were performed.

**Results:**

Forty-four per cent (113/254) of pwMS were dissatisfied with their treatment status and 53% (135/254) had DC. DC (*p* = 0.013) and decisional regret (*p* = 0.027) increase in treatment-naïve pwMS and also in those ‘offered treatment’ dissatisfied with their treatment status (*p* < 0.0001), whilst those ‘on treatment’ have low Decisional Regret Scale (DRS) score (*p* = 0.0005). DC and DRS were only correlated with treatment status in those on treatment and not in treatment-naïve patients. F (58/135) pwMS satisfied with treatment had DC. DC (*n* = 236, adjusted *R*^2^ 0.137, *p* = 0.000) and DRS (*n* = 235, adjusted *R*^2^ 0.232, *p* = 0.000) were increased by dissatisfaction with treatment, lower potency treatment, being from the ‘MS conference attendees’ cohort and reliance on the doctor’s decision, with DC additionally associated with being employed.

**Conclusions:**

DC and decisional regret vary in populations at different stages of initiating DMTs and are impacted by non-treatment issues.

## Introduction

Patients have a right to be engaged in decisions concerning their own care. Shared Decision Making (SDM) is often cited as a desirable mechanism to achieve this, where the healthcare professional (HCP) and the patient share responsibility for agreeing a way forward.^1^ However, despite the availability of multiple tools it is not clear how the decision-making process should be measured.^2–5^ Decisional conflict (DC) refers to an individual’s perception of uncertainty about the course of action to take. DC is a continuum where an individual’s perception may change. The SURE scale is a validated modification of the DC scale which generates a binary outcome; however, it is easy to use and quick to administer, with the absence of DC indicating whether a decision has been ‘successful’.^1,6^ The Decisional Regret Scale (DRS) is an alternative measurement of decision-making outcome. The DRS is based on a 20-point scale and indicates if a negative or positive experience has informed a current decision.^7^ The Control Preference Scale (CPS) indicates a patient’s preferred role in shared treatment decision-making. This ranges from being patient-led to HCP-led, and has been used effectively in a multiple sclerosis (MS) setting.^8^

Starting disease-modifying treatments (DMTs) is a complex decision. Treatment is recommended early in the disease course^9^ when symptoms may be minimal or absent and other life goals, for example starting a family, need to be considered.^10^ Treatment comes with wide range of benefits, routes of administration and risks. This is further complicated by the increasing number of therapeutic options with limited knowledge of how they should be used in individuals and in relation to each other.^11^ These factors mean the onus for the decision is on people with MS (pwMS) and their healthcare team. Decision making is a process, and those who have not yet chosen therapy are already in the ‘process’^12^ and will be aware that decisions need to me made, such as whether to start or not and what therapy to choose. Therefore a person may have DC about the options, but also decisional regret about a course of action they did not take or are yet to take, including not starting therapy. Our aim was to determine if DC and decisional regret – specific to DMTs – were relevant in MS in those at differing stages of making decisions about DMTs.

## Methods

### Cohorts

Three cohorts of pwMS (*n* = 254) took part: a ‘MS conference attendees’ cohort consisting of pwMS who were attending a study day in September 2014 for them, their families and HCPs. They were not part of a study but were part of an audit, thus data was anonymous (Imperial College Healthcare NHS Trust neurosciences audit project). The second (‘on treatment’ cohort) consisted of pwMS who were part of an established research study (NRES: 09/H0708/61). The primary goal of the study was to facilitate patient access to clinical trials testing new therapeutic interventions, or access to second-line treatments. All participants were on therapy and were not actively seeking a change in treatment when contacted. A respondent was not selected if ≥2 years had elapsed since their last known clinical appointment and subjects were posted anonymised questionnaires in January 2015. The third (‘offered treatment’ cohort) consisted of pwMS reviewing treatment options at outpatient clinics between April 2016 and April 2017 as part of the Decisions Of Uncertainty Broaching Treatment in MS (MS-DOUBT) study (REC: 16/LO/0153). Patients were chosen independent of the lead researcher by neurologists; they had to have relapsing MS (RMS) or secondary progressive MS (SPMS), be aged ≥18 years and eligible for DMTs. The patient could be on or off treatment at the time.

### Questionnaires

Patients were asked a specific question with regard to their treatment status. If they were on treatment: were they happy to continue or considering changing? Or if they were not on treatment: were they considering treatment options or not? Patients were also asked to complete the following tools: the SURE scale,^6^ the DRS^7^ and the CPS.^8^ For the SURE scale measuring DC patients were asked – ‘With reference to treatment, which of the following options best reflects your current situation?’. Patients answering ‘no’ to one or more items (SURE total score ≤3) have clinically significant DC. For the DRS patients were asked ‘Based upon your current treatment status (even if you are not on treatment), please show how you feel about these statements’. The DRS consists of five items with a five-point Likert scale giving a score between 0 (no decisional regret) to 100 (highest decisional regret).^7^ The CPS^8^ comprises five scenarios involving treatment decision-making and indicates a patient’s preferred role in shared treatment decision-making. Each scenario presents a different cartoon and statement including a preference ranging from an active, autonomous role, sharing the decision with physician, through to a passive role whereby the physician leads on the decision. Here, the CPS was administered in an amended form (with permission of the lead author – Solari), whereby users were asked to pick their main preference from five patient-physician scenarios. This was because the questionnaires were not administered by the investigator and were completed independently by the participant; patients were asked to pick their preferred role from the five options with reference to their most recent consultation with a neurologist.

### Statistical analysis

The following information was obtained. MS type: relapsing–remitting (RR)MS, SP/primary progressive (PP) MS; how long had they been diagnosed: 0–3 years, ≥4 years; sex; age group 18–44, 45 and above; ethnicity: white (all) or other (all) incorporating black, Asian, mixed & all other groups (due to smaller numbers it was not possible to stratify the ethnic groups further); marital status: with partner (married, co-habiting, civil partnership, single (separated, divorced, single); employment status: employed: full/part-time, self-employed or other state of employment, or not in employment: disabled, retired, homemaker, unemployed, student or other. Subjects were asked to choose one of four options to categorise them into two groups by treatment status: ‘satisfied’: on or off treatment but satisfied with current status; or ‘not satisfied’: on or off treatment and considering options; treatment-naïve patients or with treatment history; cohorts coded as ‘MS conference attendees’, ‘on treatment’, ‘offered treatment’. CPS was classified as Active, Active-Collaborative, Collaborative, Passive-Collaborative, Passive. SURE groups were classified as DC: yes or no. Treatment potency was classified as no treatment, moderate or high potency, as defined by the Association of British Neurologists criteria.^14^ Items 2 and 4 of the DRS were reverse coded as per the creator’s instructions; a higher number is indicative of more regret. Scores were converted to a 0–100 scale by subtracting 1 from each item then multiplying by 25. To obtain a final score, the items were summed and averaged. A score of 0 means no regret; a score of 100 means high regret.^7^

Data is presented as ratios, percentages and mean and standard deviation where appropriate. Statistical analysis was performed using the paired *t*-test, two-way ANOVA (GraphPad Prism, version 7.02 September 2016: www.graphpad.com). Categorical frequency data was analysed using Chi-Square and Fishers exact test (Vassarstats: www.vassarstats.net accessed 04/02/2018) where appropriate. Modelling the dependence of the three scores (DC, DRS and CPS) on the covariates was performed using logistic regression models using R (version 3.4.2: 28-09-2017). Covariates were described as odds ratios, reported with 95% confidence intervals and *p*-values testing the null hypothesis of no effect. Graphs were drawn using (GraphPad Prism, version 7.02 September 2016: www.graphpad.com).

## Results

### Population characteristics

In total, 105/116 responses obtained from the ‘MS conference attendees’ cohort were complete. Some 169 pwMS were sent questionnaires in the ‘on treatment’ cohort; 78 responded (46% response rate) of which two responses were incomplete. As part of the ‘offered treatment’ cohort, 129 pwMS were approached and 73 responded (57% response rate). A total of 254 pwMS were part of the total analysis (73% female, 92% RRMS); their demographics are described in Table 1. Treatment-naïve subjects were derived from the ‘MS conference attendees’ and ‘offered treatment’ cohorts (Table 1).

**Table 1.** Demographic features of the three cohorts of pwMS

Parameter	‘MS conference attendees’ (*n* = 105)			‘On treatment’(*n* = 76)			‘Offered treatment’ (*n* = 73)			*p*-value (comparing cohorts)
Relapsing MS*	87 (85%), 2 missing			74 (100%), 2 missing			68 (94%), 1 missing			*p* = 0.0006
MS diagnosis (0–3 yrs)**	32 (30%), 3 missing			0 (0%), 5 missing			32 (46%), 4 missing			*p* < 0.0001
Treatment naïve***	14 (13%)			0 (0%)			22 (31%), 3 missing			*p* = 0.003
Treatment potency (no treatment (0), moderate (1), high (2))**** number on [injectable/orals]	0 = 17 (16%)	1 = 28 (27%)[17,11]	2 = 60 (57%)	0 = 11 (15%)	1 = 38 (50%)[11, 27]	2 = 27 (35%)	0 = 39 (53%)	1 = 30 (41%)[7, 22]	2 = 4 (6%)	*p* = 0
Male sex	31 (30%)			20 (26%)			17 (23%)			NS
Age 18–44 years	48 (46%)			48 (63%)			40 (55%)			NS
White ethnicity	89 (85%)			58 (77%), 1 missing			59 (82%), 1 missing			NS
With partner	76 (72%)			50 (66%)			33 (52%), 10 missing			NS
Employed	56 (53%)			48 (64%), 1 missing			45 (68%), 7 missing			NS

*Differences in the ratios of MS type (PPMS/SPMS & RMS) between the groups (*p* = 0.0006) was due to SPMS/PPMS participants being excluded from the ‘on treatment’ and ‘offered treatment’ cohorts as a result of their study entry criteria. **There was a higher proportion of newly diagnosed (0–3 yrs) pwMS in the ‘offered treatment’ than the ‘MS conference attendees’ cohort (*p* = 0.046) and the ‘MS conference attendees’ cohort had a higher proportion of newly diagnosed pwMS than the ‘on treatment’ cohort (p =  < 0.0001).

***There were in total 36 (14%) treatment-naïve pwMS, none in the ‘on treatment’ cohort, significantly less than the ‘MS conference attendees’ cohort (14/105 [13%], *p* = 0.0009), and the ‘offered treatment’ cohort (22/70 [31%], *p* =  < 0.0001). There were significantly more treatment-naïve pwMS in the ‘considering treatment’ versus the ‘MS conference attendees’ cohort (*p* = 0.003).

****We compared only the moderate and high-potency treatment groups and found a significant difference (2 × 3 Fisher’s Exact Test, *p* = 0) confirming that the ‘MS conference attendees’ cohort had a higher percentage on high-potency treatment. This cohort also had the lowest percentage of treatment-naïve pwMS. NS – not significant

### Treatment-naïve pwMS and those ‘offered treatment’ have high levels of dissatisfaction with their current treatment status

The treatment status ‘not satisfied’ was found in 44% (113/254) of the total population; 33% (35/105) of the ‘MS conference attendees’ cohort, 25% (19/76) of the ‘on treatment’ cohort and 81% (59/73) of the ‘offered treatment’ cohort. This was significantly higher in the latter (chi-square, *p* < 0.0001), consistent with a decision needing to be made. Treatment status ‘not satisfied’ was also high in those who were treatment naïve, where 26/36 (72%) were ‘not satisfied’ with their current treatment or lack of treatment. Notably, a majority were in the ‘offered treatment’ cohort, and a multivariate analysis of the total population using the initial factors of age, gender, ethnicity, employment status, marital status, type of MS, time from diagnosis, treatment naïve and cohort found that only being from the ‘offered treatment’ cohort (1.608 [1.408, 1.836], *p* < 0.0001 (odds ratio [95%CI: upper, lower], *p*) was associated with being ‘not satisfied’ with treatment status (adjusted *R*^2^ 0.214, *n* = 254, *p* < 0.0001).

### Treatment-naïve pwMS have high levels of DC and decisional regret whereas those ‘on treatment’ have low levels of decisional regret

**Figure 1.**
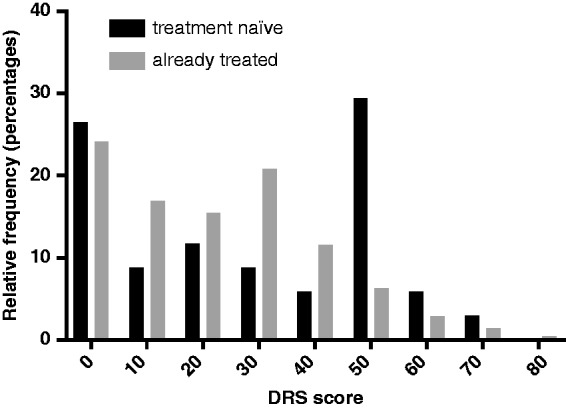
The DRS scores patients who are treatment naïve versus those who were on of who had been on treatment. There is a significant difference between the distributions of DRS scores in the treatment-naïve cohort (*n* = 36, three questionnaires not completed) versus those who were or who had been on treatment (*n* = 215, three questionnaires not completed) (Kolomogorov–Smirnov test, *p* = 0.027).

**Figure 2.**
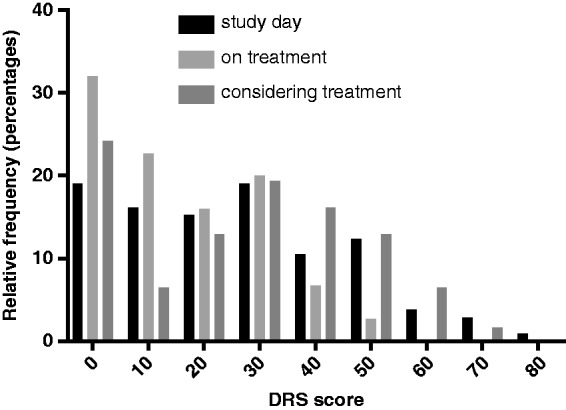
The ‘MS conference attendees’ cohort had the highest decisional regret compared with the ‘on treatment’ cohort. Notably the ‘offered treatment’ cohort had a lower DRS as many had not been on treatment.

In the total population 53% (135/254) of pwMS were found to have DC. This was significantly increased to 27/36 (75%, *p* = 0.013) in the treatment-naïve group. 59% (62/105) of the ‘MS conference attendees’ cohort had DC, 53% (39/73) in the ‘offered treatment’ cohort and 45% (34/76) of the ‘on treatment’ cohort. There were no significant differences between the cohorts. There was a peak of high DRS in the treatment-naïve group compared with those who were or had been on treatment (Figure 1). There was a difference between the cohorts in terms of their decisional regret (Figure 2, Kruskal–Wallis, *p* = 0.0005), with the ‘on treatment’ cohort having significantly less decisional regret than the ‘MS conference attendees’ (*p* = 0.0028) and ‘offered treatment’ cohort (*p* = 0.0016).

### Highest levels of DC in pwMS ‘not satisfied’ with current status are seen in ‘offered treatment’ cohort

Thirty per cent (77/254) had both DC and dissatisfaction with their treatment status; 30% (31/105) of the ‘MS conference attendees’ cohort, 18% (14/76) of the ‘on treatment’ cohort and 44% (32/73) of the ‘offered treatment’ cohort. We found that the ‘offered treatment’ group ‘not satisfied’ with their treatment had significantly higher DC compared with those ‘on treatment’ (*p* = 0.000) and the ‘MS conference attendees’ cohort (*p* = 0.049), whereas there was a trend for a difference between the ‘MS conference attendees’ and ‘on treatment’ groups (*p* = 0.088). In the total treatment-naïve population, 72% (26/36) were not satisfied and recorded DC. Confirming that DC is affected by other factors, 58/135 (43%) had DC but were satisfied with their treatment status.

### Treatment satisfaction and DC and decisional regret are only correlated in those who are on treatment, not in those who are treatment naïve

Despite this being a cross-sectional study, we looked further at whether treatment satisfaction and DC and/or decisional regret were correlated in those who had not yet started treatment, e.g. treatment-naïve and in those who were on treatment. As expected in those who were treatment naïve, treatment satisfaction was not correlated with DC or DRS (*n* = 34, adjusted *R*^2^ -0.015, *p* = 0.48). However, in those who were on treatment, treatment status was correlated independently (*n* = 210, adjusted *R*^2^ 0.165, *p* = 0.000) with both DC (1.161 [1.020, 1.322], 0.024) and decisional regret (1.009 [1.006, 1.013], 0.000).

### DC and decisional regret in the total population are increased by dissatisfaction with treatment, lower potency treatment, being employed and more reliance on the doctor’s decision

To gain further insight into factors that may influence DC and DRS, we performed a multivariate analysis with the following covariates: age, sex, ethnicity, employment, marital status, MS disease type, time from diagnosis, treatment status, cohort and treatment potency. Five variables were associated with DC (*n* = 245, adjusted *R*^2^ 0.142, *p* = 0.000; Table 2, column 1) and four variables were associated with higher DRS (*n* = 241, adjusted *R*^2^ 0.222, *p* = 0.000; Table 2, column 3). Having DC and decisional regret were both associated with being from the ‘MS conference attendees’ cohort, being on a lower potency treatment, dissatisfaction with treatment and being of non-white ethnicity. In addition, DC was associated with being employed. Though there was a correlation between disease duration and employment status in the total cohort (47/63 employed and disease duration of >4 years vs. 96/173 (75%) employed and disease duration 0–3 yrs; chi-square *p* = 0.010), disease duration itself was not associated with DC.

**Table 2.** Multivariate analysis of factors associated with DC, DRS and CPS with the factors: ethnicity, employment, treatment status, cohort, MS type and treatment potency.

Factor	Odds ratio (95%CI upper, lower), *p*				
	DC	DC with CPS instead of ethnicity	DRS	DRS with CPS instead of ethnicity	CPS
Treatment status	1.253 (1.087, 1.444), 0.002	1.224 (1.077, 1.437), 0.003	48728 (321.1, 7.7.39 × 10^6^), 0.000	65248 (417.1, 1.02 × 10^7^), 0.000	–
CohortReference is c1 unknown Rx	Cohort 2. 0.841 (0.730, 0.970), 0.017Cohort 3. 0.724 (0.613, 0.855), 0.000	Cohort 2. 0.845 (0.729, 0.979), 0.025Cohort 3. 0.724 (0.612, 0.857), 0.000	Cohort 2. 1.6 × 10^−5^ (1.1 × 10^−7^, 0.002), 0.000Cohort 3. 0.0005 (1.2 × 10^−6^, 0.172), 0.011	Cohort 2. 1.9 × 10^−5^ (1.1 × 10^−7^, 0.003), 0.000Cohort 3. 3.9 × 10^−4^, (1.0 × 10^−6^, 0.148), 0.010	–
Employment	1.173 (1.039, 1.323), 0.010	1.186 (1.047, 1.343), 0.007	–	–	–
MS disease type	–	–	–	–	0.612 (0.412, 0.909), 0.015
Treatment Potency	0.875 (0.800, 0.958), 0.004	0.872 (0.796, 0.956), 0.004	0.006 (0.0002, 0.157), 0.002	0.007 (0.0003, 0.163), 0.002	–
Ethnicity	1.192 (1.023, 1.389), 0.024	NA	860.093(3.837, 1.9 × 10^5^), 0.015	NA	1.616 (1.210, 2.156), 0.001
CPS	NA	1.093 (1.022, 1.170), 0.010	NA	77.67 (7.089, 851), 0.0004	NA

DC (column 1) was associated with less satisfaction with treatment, being part of the ‘MS conference attendees’ cohort, being of non-white ethnicity, being in employment and on a less potent treatment. High levels of decisional regret (column 3) was associated with being less satisfied with treatment, being part of the ‘MS conference attendees’ cohort, being of non-white ethnicity and being on a less potent treatment. Higher CPS (column 5), e.g. more passivity in decision-making, was associated with non-white ethnicity and RRMS phenotype. When CPS replaced ethnicity as a variable it was then significant in the model (DC - column 2; DRS – column 4)

**Figure 3.**
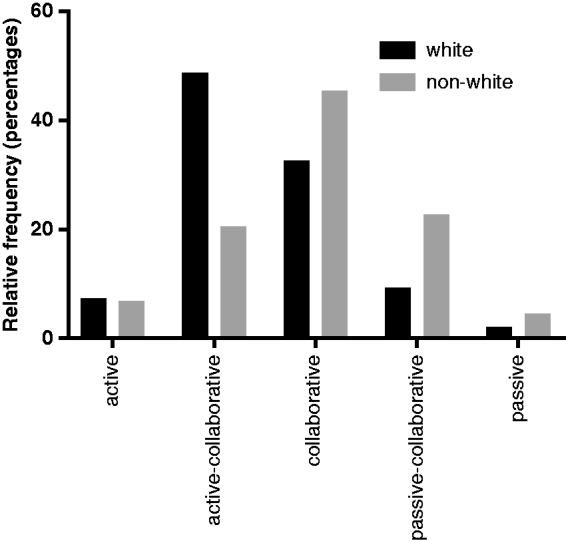
Distribution (% of total) for each category of the CPS score from the total population. Non-white ethnicity scored significantly higher CPS scores (representing a passive role) compared with white ethnicity (Kolomogorov–Smirnov test, *p*=0.006).

A similar multivariate analysis was performed for the CPS, using the same initial variables as for DC and DRS. In contrast, more passivity was associated with non-white ethnicity and having RRMS disease type (*n* = 233, adjusted *R*^2^ 0.064, *p* = 0.000; Table 2, column 5). The role of ethnicity is illustrated in Figure 3, where being of non-white ethnicity is associated with more passivity (Kolomogorov–Smirnov test, *p* = 0.006). When we added CPS to the models predicting DC (*n* = 236, adjusted *R*^2^ 0.137, *p* = 0.000; Table 2; column 2) and DRS (*n* = 235, adjusted *R*^2^ 0.232, *p* = 0.000; Table 2; column 4), CPS was a significant factor for both DC and DRS and in both cases ethnicity became non-significant. This implied that higher CPS, e.g. more reliance on the doctor’s decision rather than ethnicity, was associated with more DC and decisional regret.

## Conclusions

Here we show that DC and decisional regret are higher in treatment-naïve pwMS and DC is increased in those ‘offered treatment’ dissatisfied with their current treatment status, whereas those ‘on treatment’ have low decisional regret. This implies that treatment has an association with lower DC and decisional regret, and was confirmed in a multivariate analysis in the total population where DC and decisional regret were increased by dissatisfaction with treatment, lower potency treatment, being employed, being from the ‘MS conference attendees’ cohort and having more reliance on the doctor’s decision. Furthermore, we show that a correlation between treatment satisfaction and DC/decisional regret is only present in those who have been exposed to treatments and not before, e.g. treatment-naïve patients.

We included three different populations of pwMS to try and determine the role of treatment in decision-making about DMTs in MS. The combined populations showed a high level of dissatisfaction (44%) with their treatment status (on or off treatment), with highest levels in the treatment-naïve subgroup. On a cohort basis, this was highest in those ‘offered treatment’ and lowest in those ‘on treatment’. This is not surprising, as the ‘offered treatment’ cohort had just come from a consultation where it was made clear there were decisions to be made, whereas the ‘on treatment’ cohort did not make active contact to discuss therapy.

The majority of the total population had DC (53%). However, in contrast to dissatisfaction with treatment, DC was highest in those from the ‘MS conference attendees’ cohort – significantly above the ‘on treatment’ population. This was increased further when we studied those in each cohort who were not satisfied with their current treatment status. The high levels seen in the ‘MS conference attendees’ cohort is interesting in that this group were attending an event aiming to inform about MS therapies. That they were part of an anonymous audit and were not part of a study highlights a potential issue in understanding the aetiology of DC. Firstly, their attendance at a study day with DC indicates actively seeking knowledge possibly to resolve DC – however, we were not able to contact them directly to confirm this. Secondly, in-depth studies may be biased as a result of not engaging sections of the MS population. Again, the low levels of DC found in the population contacted at home who have not sought out nor attended clinic where issues of treatment would have been raised is not surprising. Reassuringly, our findings using the DRS scale essentially reflect the DC findings, further validating the results.

Here we have related DC and decisional regret to the process of starting DMTs in MS to determine if and how they change when DMTs are started. We have done this by relating to specific questions about a patient’s current status with regard to treatments: either satisfied with what they are on, or that they are not on treatment in the case of those who are treatment naïve. DC and decisional regret are influenced by multiple factors, and indeed we see high DC and decisional regret in treatment-naïve patients, but it is not correlated with treatment satisfaction, where DC and DRS is correlated with treatment satisfaction in those on treatment.

The quantitative multivariate analysis performed across the whole population highlighted other factors associated with DC and decisional regret; this included being on lower potency treatment, which was still evident when the treatment-naïve group was removed and more passive involvement in decision making, whilst being in employment was associated with higher DC alone. The association with lower DC and decisional regret in those on treatment and higher potency treatment reinforces the finding that treatment is associated with reduced DC. It may relate to stronger treatments having greater beneficial impact on quality of life (QoL),^15^ or it could also relate to reduced day-to-day side effects associated with high-potency therapies.^16^ This is an important issue for HCPs to be aware of, as there may be a desire amongst pwMS to access higher potency treatments to achieve the best possible outcomes. We initially identified non-white ethnicity as a potential factor influencing DC and decisional regret. As has been seen previously in multiple populations, we found that a more passive role preference was related to ethnicity.^17–19^ Consistent with this, when CPS was added to the factors associated with DC and decisional regret, ethnicity became non-significant, implying that a more passive role preference was associated with more DC and decisional regret. Increased patient involvement and SDM has decreased DC in other conditions, and in turn lower DC had a favourable influence of patient satisfaction with the HCP.^20,21^ This supports involvement of the patient during the clinical encounter, but whether this is realised depends on the perception of the patient. Unexpectedly, we also found that being in employment was associated with DC. Some studies have associated unemployment with a prolonged disease duration,^22^ but we did not find disease duration to be independently associated with DC and DRS. The association may occur through a confounder not measured here, such as fatigue.^23^

This study has a number of limitations. Decision-making is a process, and many factors influence DC and decisional regret; as a result, it is necessarily imperfect concentrating the measured DC and DRS on a decision to start treatments. Furthermore, DC is not a binary response as measured here, and we may have not captured the many facets of DC here. Finally, this study is cross-sectional, thus differences we have seen in those who are treatment naïve and on treatment need to be replicated longitudinally.

Earlier work implied that DC was not involved in the decision-making process;^24^ however, this was in the context of a randomised controlled trial (RCT), whereas here we find those not in direct contact with HCPs at a study day have the highest DC, implying encountering HCPs and being involved in an RCT in itself could resolve many issues driving DC.^25,26^ Not unexpectedly, there are additional factors driving DC not directly associated with treatment, and these require further characterisation. Decision-making is a continuous process and it is necessary to extend these findings into a prospective study, as interaction between perceived disease and treatment risk evolves over time. However, this work offers DC and decisional regret as potential outcome measures to quantify the impact of decisions on pwMS.

## Supplemental Material

Supplemental material for Initiating disease-modifying treatments in multiple sclerosis: Measuring the decision process using decisional conflict and decisional regret scales Click here for additional data file. Supplemental Material for Initiating disease-modifying treatments in multiple sclerosis: Measuring the decision process using decisional conflict and decisional regret scales by DD Wilkie, A Solari and R Nicholas in Multiple Sclerosis Journal—Experimental, Translational and Clinical
