# Supplementary material for: Initiating disease-modifying treatments in multiple sclerosis: Measuring the decision process using decisional conflict and decisional regret scales
Source: Mult Scler J Exp Transl Clin. 2019 Feb 27;5(1):2055217319833006. doi: 10.1177/2055217319833006 (PMC6393834; doi:10.1177/2055217319833006)
Supplement: Supplemental material for Initiating disease-modifying treatments in multiple sclerosis: Measuring the decision process using decisional conflict and decisional regret scales [file Supplemental_Material.pdf]

Appendix 1. Questionnaires

1.1 Demographic questionnaire

**What is your gender?**	1. Male ☐	2. Female ☐
**What is your age?**	1. 18 to 24 ☐<br>2. 25 to 34 ☐<br>3. 35 to 44 ☐<br>4. 45 to 54 ☐<br>5. 55 to 64 ☐<br>6. 65 to 75 ☐<br>7. 75 or older ☐	
**What is your ethnicity?**	1. White - British ☐<br>2. White - Irish ☐<br>3. Any other white background ☐<br>4. Mixed – White & Black Caribbean ☐<br>5. Mixed – White & Black African ☐<br>6. Mixed – White & Asian ☐<br>7. Any other mixed background ☐<br>8. Asian or Asian British - Indian ☐	9. Asian or Asian British - Pakistani ☐<br>10. Asian or Asian British - Bangladeshi ☐<br>11. Any Other Asian Background ☐<br>12. Black or Black British - Caribbean ☐<br>13. Black or Black British - African ☐<br>14. Any Other Black background ☐<br>15. Chinese ☐<br>16. Any other ethnic group (please specify) ☐ ________________
**Marital Status**	1. Single ☐<br>2. Married ☐	5. Co-habiting ☐<br>6. Civil partnership☐

	3. Divorced ☐<br>4. Separated ☐	7. Same-sex marriage ☐<br>8. Widowed ☐
**Which of the following best describes your employment status?**	1. Employed ☐<br>2. Home maker ☐<br>3. Retired ☐<br>4. Student ☐<br>5. Unemployed ☐<br>6. Other circumstances (please state) _______________	
**Type of MS**	1. Relapsing-Remitting ☐<br>2. Secondary Progressive ☐<br>3. Primary Progressive ☐<br>4. Clinically Isolated Syndrome (MS not diagnosed) ☐<br>5. Not known ☐	
**Year of MS Diagnosis (if known)**	1. Please state_________<br>2. Don't know ☐	

1.2 Treatment Potency

The following table lists MS treatments currently available either licensed or in development (eg. as part of a clinical trial). For each treatment, please tick one answer.

1. Natalizumab (Tysabri)	1. Currently receiving this treatment ☐	2. Have tried this treatment in the past but now stopped ☐	3. I have knowledge of this treatment but have never been prescribed it ☐	4. I have no knowledge of this treatment and have never been prescribed it ☐	5. I am either on this treatment or placebo as part of a clinical trial ☐

2. Fingolimod (Gilenya)	1. Currently receiving this treatment ☐	2. Have tried this treatment in the past but now stopped ☐	3. I have knowledge of this treatment but have never been prescribed it ☐	4. I have no knowledge of this treatment and have never been prescribed it ☐	5. I am either on this treatment or placebo as part of a clinical trial ☐
3 Alemtuzumab (Lemtrada)	1. Currently receiving this treatment ☐	2. Have tried this treatment in the past but now stopped ☐	3. I have knowledge of this treatment but have never been prescribed it ☐	4. I have no knowledge of this treatment and have never been prescribed it ☐	5. I am either on this treatment or placebo as part of a clinical trial ☐
4. Beta interferon (eg. Avonex, Rebif, Betaferon, Extavia)	1. Currently receiving this treatment ☐	2. Have tried this treatment in the past but now stopped ☐	3. I have knowledge of this treatment but have never been prescribed it ☐	4. I have no knowledge of this treatment and have never been prescribed it ☐	5. I am either on this treatment or placebo as part of a clinical trial ☐
5. Dimethyl Fumerate (Tecfidera/BG-12)	1. Currently receiving this treatment ☐	2. Have tried this treatment in the past but now stopped ☐	3. I have knowledge of this treatment but have never been	4. I have no knowledge of this treatment and have never been	5. I am either on this treatment or placebo as part of a clinical trial ☐

			prescribed it ☐	prescribed it ☐	
6. Glatiramer Acetate (Copaxone)	1. Currently receiving this treatment ☐	2. Have tried this treatment in the past but now stopped ☐	3. I have knowledge of this treatment but have never been prescribed it ☐	4. I have no knowledge of this treatment and have never been prescribed it ☐	5. I am either on this treatment or placebo as part of a clinical trial ☐
7. Teriflunomide (Aubagio)	1. Currently receiving this treatment ☐	2. Have tried this treatment in the past but now stopped ☐	3. I have knowledge of this treatment but have never been prescribed it ☐	4. I have no knowledge of this treatment and have never been prescribed it ☐	5. I am either on this treatment or placebo as part of a clinical trial ☐
8. Laquinimod	1. Currently receiving this treatment ☐	2. Have tried this treatment in the past but now stopped ☐	3. I have knowledge of this treatment but have never been prescribed it ☐	4. I have no knowledge of this treatment and have never been prescribed it ☐	5. I am either on this treatment or placebo as part of a clinical trial ☐
9. Stem Cells	1. Currently receiving this treatment ☐	2. Have tried this treatment in the past but	3. I have knowledge of this treatment but have	4. I have no knowledge of this treatment and have	5. I am either on this treatment or placebo as

		now stopped ☐	never been prescribed it ☐	never been prescribed it ☐	part of a clinical trial ☐
10. Azathioprine	1. Currently receiving this treatment ☐	2. Have tried this treatment in the past but now stopped ☐	3. I have knowledge of this treatment but have never been prescribed it ☐	4. I have no knowledge of this treatment and have never been prescribed it ☐	5. I am either on this treatment or placebo as part of a clinical trial ☐
11. IVIg (intravenous innumoglobulin)	1. Currently receiving this treatment ☐	2. Have tried this treatment in the past but now stopped ☐	3. I have knowledge of this treatment but have never been prescribed it ☐	4. I have no knowledge of this treatment and have never been prescribed it ☐	5. I am either on this treatment or placebo as part of a clinical trial ☐
12. Mitoxantrone	1. Currently receiving this treatment ☐	2. Have tried this treatment in the past but now stopped ☐	3. I have knowledge of this treatment but have never been prescribed it ☐	4. I have no knowledge of this treatment and have never been prescribed it ☐	5. I am either on this treatment or placebo as part of a clinical trial ☐

13. Cyclophosphamide	1. Currently receiving this treatment ☐	2. Have tried this treatment in the past but now stopped ☐	3. I have knowledge of this treatment but have never been prescribed it ☐	4. I have no knowledge of this treatment and have never been prescribed it ☐	5. I am either on this treatment or placebo as part of a clinical trial ☐
14. Zenapax (Daclizumab)	1. Currently receiving this treatment ☐	2. Have tried this treatment in the past but now stopped ☐	3. I have knowledge of this treatment but have never been prescribed it ☐	4. I have no knowledge of this treatment and have never been prescribed it ☐	5. I am either on this treatment or placebo as part of a clinical trial ☐
15. Ocrelizumab	1. Currently receiving this treatment ☐	2. Have tried this treatment in the past but now stopped ☐	3. I have knowledge of this treatment but have never been prescribed it ☐	4. I have no knowledge of this treatment and have never been prescribed it ☐	5. I am either on this treatment or placebo as part of a clinical trial ☐

1.3 Treatment Status

Question asked: 'With reference to treatment, which of the following options best reflects your *current* situation?'

Please tick one box.

1. Satisfied to continue with existing treatment ☐	2. On treatment but considering a treatment switch ☐	3. Not on treatment but considering treatment options ☐	4. Not on treatment and not considering treatment ☐

1.4 DC (SURE scale)

Further to the answer you gave (above) referencing treatment choice:

1. Do you feel sure about the best choice for you?	1. Yes ☐	2. No ☐
2. Do you know the benefits and risks of each option?	1. Yes ☐	2. No ☐
3. Are you clear about which benefits and risks matter most to you?	1. Yes ☐	2. No ☐
4. Do you have enough support and advice to make a choice?	1. Yes ☐	2. No ☐

1.5 Decisional Regret Scale

Based upon your current treatment status (even if you are not on treatment), please show how you feel about these statements by selecting a single options from each row of statements below:

1. It was the right decision	1 Strongly Agree ☐	2 Agree ☐	3 Neither Agree Nor Disagree ☐	4 Disagree ☐	5 Strongly Disagree ☐
2. I regret the choice that was made	1 Strongly Agree ☐	2 Agree ☐	3 Neither Agree Nor Disagree ☐	4 Disagree ☐	5 Strongly Disagree ☐

3. I would go for the same choice if I had to do it over again	1 Strongly Agree ☐	2 Agree ☐	3 Neither Agree Nor Disagree ☐	4 Disagree ☐	5 Strongly Disagree ☐
4. The choice did me a lot of harm	1 Strongly Agree ☐	2 Agree ☐	3 Neither Agree Nor Disagree ☐	4 Disagree ☐	5 Strongly Disagree ☐
5. The decision was a wise one	1 Strongly Agree ☐	2 Agree ☐	3 Neither Agree Nor Disagree ☐	4 Disagree ☐	5 Strongly Disagree ☐

1.6 Control Preference Scale

<u>Which of the following scenarios best fits how you would usually reach a decision about treatment either now or in the past?</u>

A - ☐ B - ☐ C - ☐ D - ☐ E - ☐

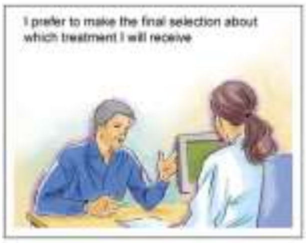

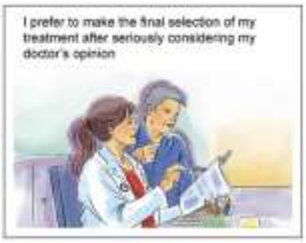

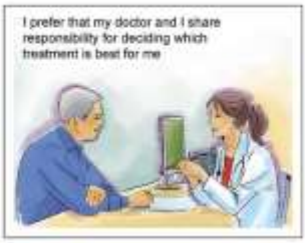

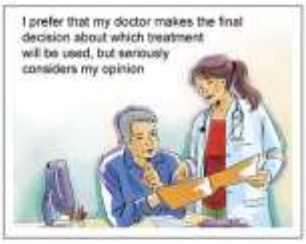

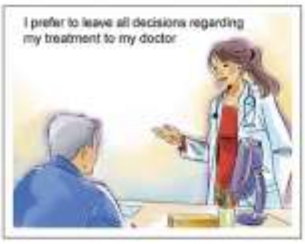

This marks the end of the questionnaire. Please kindly double-check that you have answered every question as described.

Once again, thank you for taking the time to complete the research.

References

Légaré F, Kearing S, Clay K, Gagnon S, D'Amours D, Rousseau M, O'Connor AM. Are you SURE? Assessing patient decisional conflict with a 4-item screening test. Can Fam Physician 2010; 56:e308-314.

O'Connor AM. User Manual – Decision Regret Scale [document on the Internet]. Ottawa: Ottawa Hospital Research Institute; © 1996 [modified 2003; cited 2014]. 3 p. Available from http://decisionaid.ohri.ca/docs/develop/User_Manuals/UM_Regret_Scale.pdf

Solari A, Giordano A, Kasper J, Drulovic J, van Nunen A, et al. (2013) Role Preferences of People with Multiple Sclerosis: Image-Revised, Computerized Self-Administered Version of the Control Preference Scale. PLoS ONE 8(6): e66127. doi:10.1371/journal.pone.0066127

GIORDANO, A., MATTAROZZI, K., PUCCI, E., LEONE, M., CASINI, F., COLLIMEDAGLIA, L. & SOLARI, A. 2008. Participation in medical decision-making: Attitudes of Italians with multiple sclerosis. *Journal of the Neurological Sciences,* 275**,** 86-91.

HEESEN, C., KASPER, J., SEGAL, J., KOPKE, S. & MUHLHAUSER, I. 2004. Decisional role preferences, risk knowledge and information interests in patients with multiple sclerosis. *Mult Scler,* 10**,** 643-50.
